# Promotion of Poststroke Motor-Function Recovery with Repetitive Transcranial Magnetic Stimulation by Regulating the Interhemispheric Imbalance

**DOI:** 10.3390/brainsci10090648

**Published:** 2020-09-18

**Authors:** Xiaoxia Yuan, Yuan Yang, Na Cao, Changhao Jiang

**Affiliations:** 1 Beijing Key Laboratory of Physical Fitness Evaluation and Technical Analysis, Capital University of Physical Education and Sports, Beijing 100089, China; yuanxiaoxia2019@cupes.edu.cn; 2 The Center of Neuroscience and Sports, Capital University of Physical Education and Sports, Beijing 100089, China; 3 College of Physical Education and Sports, Beijing Normal University, Beijing 100875, China; yangyuan2020@mail.bnu.edu.cn; 4 Department of Life Sciences, Graduate School of Arts and Sciences, The University of Tokyo, Tokyo 153-8902, Japan; caona@g.ecc.u-tokyo.ac.jp

**Keywords:** repetitive transcranial magnetic stimulation, stroke, motor function

## Abstract

Repetitive transcranial magnetic stimulation (rTMS) is a noninvasive brain-stimulation technique that transiently modulates cerebral cortex excitability, achieving overall positive results in poststroke motor-function recovery. Excessive inhibition of the ipsilesional-affected hemisphere by the contralesional-unaffected hemisphere has seriously hindered poststroke motor-function recovery. Hence, intracortical disinhibition can be used as an approach to managing poststroke brain injury. This technique promotes neural plasticity for faster motor-function recovery. rTMS relieves unilateral inhibition of the brain function by regulatinga interhemispheric-imbalanced inhibition. This paper summarized 12 studies from 2016 to date, focusing on rTMS on motor function after acute and chronic stroke by regulating the interhemispheric imbalance of inhibitory inputs. Although rTMS studies have shown promising outcomes on recovery of motor functions in stroke patients, different intervention methods may lead to discrepancies in results. A uniform optimal stimulus model cannot routinely be used, mainly due to the stimulus schemes, stroke types and outcome-measuring differences among studies. Thus, the effect of rTMS on poststroke motor-function recovery should be investigated further to standardize the rTMS program for optimal poststroke motor-function recovery. More randomized, placebo-controlled clinical trials with standardized rTMS protocols are needed to ensure the effectiveness of the treatment.

## 1. Introduction

Stroke caused by brain hypoxia and nutritional deficiency can damage to brain tissues and cause a series of symptoms such as motor disability, depression, cognitive impairment [1]. Motor-function deficits usually occur on unilateral body parts and are closely related to the extent of the brain injury. According to the poststroke time process, 1–3 weeks after stroke occurrence indicates the acute stage; from three weeks to six months, the subacute stage; and >six months, the chronic stage. A longitudinal study reported a significant association between hand function and corticospinal integrity within three weeks poststroke. However, cortical-network reorganization generally starts to restore lost motor functions three months after a stroke [2]. Through the recovery process, neural plasticity is considered as the primary mechanism to induce motor-function recovery, by gradually strengthening the connection among neural networks and between the brain and muscle.

Transcranial magnetic stimulation (TMS) to the brain applies a pulsed magnetic field to a target cortical area such as the motor cortex. TMS produces an induction current that affects brain metabolism and neural electrical activity. Repetitive TMS (rTMS) refers to the repeated application of a single transcranial magnetic pulse with a high-intensity magnetic field to stimulate a focused brain region that depolarizes the local neurons and produces an excitatory action potential, which excites or inhibits a population of cortical neurons depending on the stimulating protocol [3,4]. In conventional rTMS protocols, the high-frequency rTMS (HF-rTMS) uses a frequency >3 Hz, which generates an excitatory effect, whereas the low-frequency rTMS (LF-rTMS) uses <3 Hz to generate an inhibitory effect [5]. Because of its effect on regulating excitability of the cortex, rTMS is considered as a therapeutic technique for promoting motor-function recovery [6] and treatment of poststroke depression (PSD) [7,8].

The primary factors hindering poststroke functional recovery are synaptic function changes, such as decreased excitability of the affected hemisphere and interhemispheric imbalance of inhibition [9] (hereafter interhemispheric imbalance). Excessive ipsilesional hemisphere inhibition by the contralesional hemisphere after stroke seriously hinders motor-function recovery because interhemispheric inhibition aggravates neurological deficits [10,11] via the transcallosal pathway [12]. After the acute period, it was found that the transcallosal inhibition in the ipsilesional hemisphere was enhanced, while the excitability in the contralesional hemisphere increased [13]. Studies in patients with chronic stroke found a positive correlation between transcallosal inhibition and motor function of the paraplegic hand [14]. Therefore, we hypothesized that contralesional hemisphere inhibition upon the ipsilesional hemisphere is associated with poststroke injury, which is the severer the injury, the stronger the inhibition. In addition, interhemispheric inhibition is considered as a therapeutic target for poststroke recovery. Recovery is promoted by reducing transcallosal inhibition in the affected hemisphere and inhibiting excitability of the unaffected hemisphere by rTMS [15].

**Figure 1:**
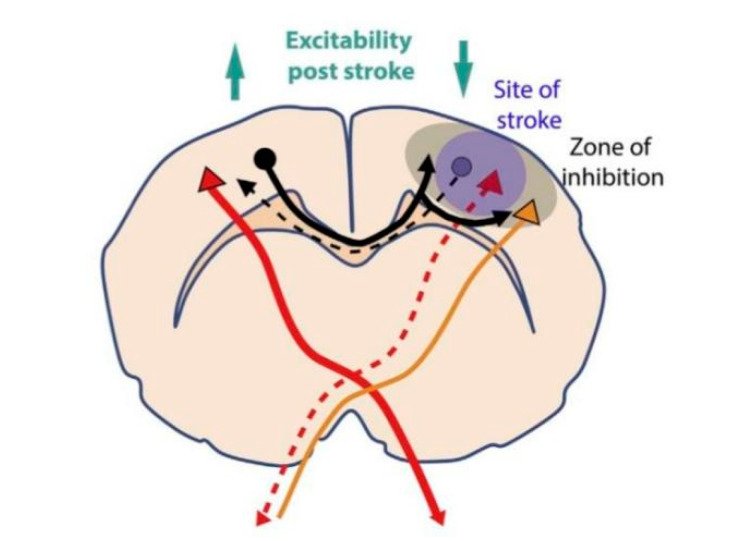
Illustration of interhemispheric imbalance post stroke in the rat [17]. The contralesional hemisphere becomes more excitable and exerts a stronger inhibition onto the ipsilesional hemisphere tissue because of unilateral cortical stroke that diminishes its inhibition to the contralesional hemisphere.

The interhemispheric imbalance model assumes unilateral brain injury after a stroke, which causes excessive inhibition by the contralesional hemisphere to the ipsilesional hemisphere disrupts the balance. Regulating the interhemispheric imbalance through noninvasive brain stimulation methods is a valuable approach for improving effects of rehabilitation after stroke (Figure 1) [16]. rTMS as a noninvasive brain stimulation can be used as such an approach for poststroke motor-function recovery because it can regulate cerebral cortex excitability and neural plasticity [17]. Depending on the different parameters of stimulation used, rTMS can induce a plasticity of excitatory synapses and changes the interhemispheric interactions. Recently, theta-burst stimulation (TBS)—as an extension of traditional rTMS—revealed encouraging application foreground in stroke. However, there is still a large space for parameter adjustment and optimization [18]. For this reason, we excluded studies that employed TBS in this review article.

In an increasing number of stroke studies, the effects of rTMS have been shown to be effective. However, the mechanisms by which rTMS enhances poststroke recovery, and thus, a more favorable outcome in stroke patients, are still unclear. Therefore, this review is aimed at evaluating the initiation of neural responses as a result of rTMS, investigating its effect on motor function among patients with different levels of stroke severity and providing an informative reference regarding usefulness of rTMS as a therapeutic tool for promoting motor-function recovery.

## 2. Materials and Methods

Because of heterogeneity of the published stroke studies, the search strategy of this review adopted a broad inclusion criteria in order to analyze a relatively large number of studies for the goal of assessing the effect of rTMS on promoting poststroke motor-function recovery.

### 2.1. Eligibility

Inclusions of studies were not limited to any particular design. The eligibility criteria were as follows: (1) rTMS was the primary intervention; (2) subjects were acute or chronic stroke patients; (3) manuscripts were written in English; (4) motor recovery outcomes of upper and lower limbs were presented separately; and (5) studies were published within a time window of January 2016 to June 2019.

### 2.2. Information Sources and Search Strategy

To carry out a systematic search for studies examined the effects of rTMS on motor recovery poststroke, terms of “repetitive transcranial magnetic stimulation”, “rTMS”, “stroke”, “acute stroke”, “chronic stroke”, “motor recovery” were searched on PubMed and Web of Science.

### 2.3. Study Selection

**Figure 2:**
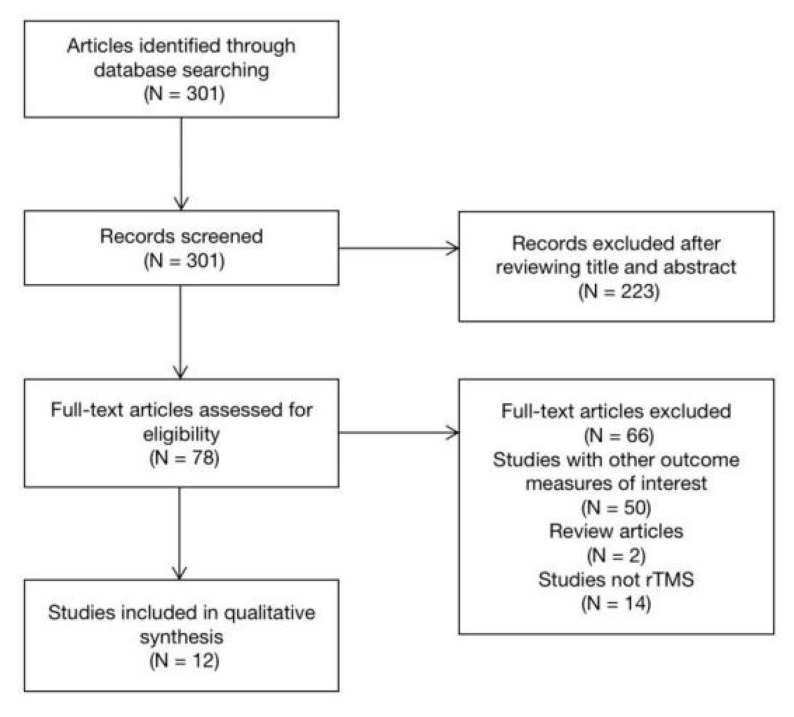
Flow diagram of the study selection process.

The second author performed the initial title and abstract search for articles that included rTMS and stroke patients. All titles and abstracts were screened, and ineligible articles were excluded. The first investigator then independently reviewed the included articles. After fully screening the articles, data were systematically extracted and summarized using Microsoft Excel to display all relevant information. Figure 2 illustrates the process of article selection.

## 3. Result

### 3.1. Interhemispheric Imbalance in Stroke: Relationship with Sensory-Motor Impairment

The interhemispheric imbalance model suggests that the excitability of the ipsilesional hemisphere is decreased and its inhibition effect on the contralesional hemisphere is weakened. Therefore, the contralesional hemisphere becomes more excitable and exert stronger inhibition on the ipsilesional hemisphere [18]. Several studies have shown that poststroke interhemispheric imbalance leads to motor dysfunction.

The motor-evoked potentials (MEP) is recorded from the target muscle as a result of stimulating the motor cortex with a single TMS (sTMS) pulse. The sTMS can quantitatively evaluate changes in corticospinal excitability and the MEP is influenced by some demographic and anthropometric indices [19,20]. Veldema et al. (2018) related severe hand dysfunction to inhibition of the corticospinal system of the ipsilesional hemisphere by evaluating the contralateral MEP. In contrast, minor hand injury showed the opposite effect; the patients’ corticospinal excitability increased in the ipsilesional hemisphere. It is worth noting that 15 of the 16 participants were acute stroke patients (≤15 days from stroke) and the remaining one was a chronic patient (>3 months from stroke) [21]. To some extent, the findings suggest that the functional recovery of the affected hand in acute-stage post stroke is associated with corticospinal system integrity and conduction pathway asymmetry.

One study found that the interhemispheric imbalance of motor cortical excitability in stroke patients was higher than that in people without stroke. The magnitude of this imbalance is related to the severity of sensorimotor injury [22]. Paired-pulse TMS (ppTMS) can test various neural circuits involved in intracortical inhibition or facilitation and interhemispheric inhibition or facilitation. Intracortical inhibition and facilitation are achieved by applying paired pulses with an inter-pulse interval (IPI) using a single coil with a short IPI (1–5 ms) leading to inhibition and a long IPI (>5 ms) facilitation. In contrast interhemispheric simulations are accomplished with double coils (one on each hemisphere) and different stimulus intensities between the pulses delivered by the two coils with a short IPI [23,24]. Seo et al. (2018) found that the contralesional hemisphere was inhibited more easily than the ipsilesional hemisphere measured by intracortical inhibition (ICI) and facilitation (ICF) among 103 acute and subacute stroke patients with moderate stroke-inflicted brain injury [25]. The ICI of patients with severe hemiparesis after an acute stroke was significantly lower than that of healthy participants when the IPI was 2–4 ms, which was related to the rapid motor-function recovery [26,27]. Furthermore, when the ppTMS was applied to the contralesional hemisphere, the ICI decreased in the patient–group.

In patients with chronic stroke, the stimulus threshold for eliciting ipsilesional ICI and contralesional ICF decreased significantly; however, there was no such difference between the cerebral hemispheres in the healthy group [28]. In addition to poststroke ICI, poststroke motor performance was related to motor cortex excitability, cerebral hemisphere connectivity and corticospinal integrity [29]. The literature suggests that the contralesional hemisphere’s over-inhibition of the ipsilesional hemisphere hinders motor-function recovery on the affected side in stroke patients. This demonstrates that interhemispheric balance and intracortical disinhibition play a significant role in ameliorating brain function in stroke patients [30].

### 3.2. rTMS on Motor-Function Recovery after Acute Stroke

rTMS that induces cortical excitatory changes can be adopted to manage poststroke motor-function recovery in clinical settings. Table 1 shows the details of studies that rTMS on motor function recovery after acute stroke. One study compared the difference between rTMS, and conventional treatment reported that the effect of 10-Hz rTMS on increasing strength of the extensor digitorum muscle in patients with ischemic stroke was similar to that of the conventional intervention group [31]. Therefore, rTMS could be a potential intervention to strengthen poststroke muscle strength recovery. 

Several studies applied LF-rTMS over the unaffected contralesional hemisphere and observed elevated excitability, indicating that intracortical excitability of the contralesional hemisphere should be suppressed to regulate the interhemispheric imbalance in stroke patients. Stimulation sites for rTMS should be selected in the intervention program considering the effects of stimulus target in the brain relevant to the injury site. The majority of the rTMS studies chose M1 as the stimulation target for poststroke motor recovery. However, other cortical areas have also been targeted for stimulation for motor recovery [32]. Matsuura et al. (2015) investigated beneficial effects of rTMS on functional recovery and electrophysiological indices in patients with acute stroke [33]. This study included an rTMS group and a control (false-rTMS) group for comparisons. The patients in the rTMS group received 1200 pulses of 1-Hz rTMS five times a day in the contralesional motor cortex. In contrast, patients in the false-rTMS group received the same number of false stimulations to the contralesional motor cortex. It was found that the refined motor activity was associated with motor potential and negative slope of the unaffected contralesional hemisphere, whereas the control group had no significant changes. LF-rTMS over the unaffected contralesional hemisphere may inhibit the excitability of the affected contralesional hemisphere, increase the neuron excitability of the motor cortex and premotor cortex (PMd) of the affected contralesional hemisphere, promote the normalization of interhemispheric imbalance and restore function of the paraplegic limbs in patients with acute stroke [33]. Furthermore, a study involving ten subacute stroke patients with moderate hand injury reported the effect of 1-Hz rTMS on hand function rehabilitation [34]. Although the same 1-Hz rTMS was applied to the patients’ affected contralesional hemisphere, the PMd was selected as the target region. This study showed that the motor function of the affected hand improved significantly with rTMS. However, the stimulation effects on motor function over the diverse brain regions should be investigated further.

Similarly, the application of HF-rTMS to the contralesional hemisphere promotes intracortical excitability to normalize interhemispheric imbalance. Investigators should focus on addressing the question of how to design stimulation methods to maximize the rehabilitation benefits of HF-rTMS. In the acute stage, inhibitory stimulation of the unaffected contralesional hemisphere is more effective than excitatory stimulation of the affected contralesional hemisphere [35]. Moreover, whether effects between inhibitory low-frequency stimulation and combined low-frequency and high-frequency stimulations on the uninjured side of the brain differ remains to be elucidated. An acute stroke study compared the high- with low-frequency rTMS differences on early upper limb motor function. In this study, 62 patients were divided randomly into the LF-rTMS, LF-HF-rTMS and control groups who receive the same conventional rehabilitation and rTMS for 15 consecutive days. The motor enhancement results in the LF-HF-rTMS group are more effective than those in the LF-rTMS and control groups at the end of treatment and the 3-month follow-up. Although both LF-HF and LF-HF-rTMS can promote effective recovery of upper extremity motor function in patients with acute stroke, the combination of HF-rTMS and LF-rTMS is more beneficial for improved motor function compared with LF-rTMS alone [36].

The validity of the results of rTMS for corticospinal pathways are mainly applicable for patients with acute stroke. Activating the encephalic region transmits signals to the muscles via the corticospinal pathway conduction, which contributes to poststroke motor execution, a prognostic indicator of poststroke motor-function recovery [37]. Gerschlager et al. 2002 [38] reported that MEPs of the right hand and forearm muscles increased significantly within 30 min after rTMS over the ipsilateral cerebellum and posterior neck, indicating that cerebellar and peripheral nerve stimulations affected the corticospinal cord excitability. The sustained effect of cerebellar rTMS on corticospinal excitability seems to be mediated by stimulating peripheral rather than central structures. Peripheral rTMS can cause long-term changes in the spinal reflexes.

### 3.3. rTMS on Motor-Function Recovery after Chronic Stroke

Table 1 shows the details of studies that rTMS on motor function recovery after chronic stroke. 

The effects of rTMS in patients with chronic stroke are similar, i.e., inhibiting the unaffected contralesional hemisphere according to the principle of interhemispheric mutual inhibition, to enhance the recovery of patients’ motor function. To verify the effects of LF-rTMS on the lower extremity function and motor neuron excitability in patients with chronic stroke, 20 chronic ischemic stroke patients with upper limb hemiparesis were divided into 1-Hz rTMS and control groups. The unaffected lower extremity motor area was stimulated, and results showed that the lower extremity motor function measured by modified Ashworth scale was significantly improved only after the real rTMS intervention. The improvement persisted after 1 week [44]. LF-rTMS over the upper limb motor area of the affected contralesional hemisphere promoted motor-function recovery of the upper limbs [41].

rTMS strengthens the motor function in patients with mild to moderate chronic stroke; however, its effect in patients with severe chronic stroke remains to be elucidated. Demirtastatlidede et al. 2015 [46] explicated that 1-Hz rTMS in the affected contralesional hemisphere may help transform the neuroplasticity of patients with severe chronic stroke. LF-rTMS-enhanced exercise training could improve the dyspraxia of patients with severe upper limb dyspraxia after a chronic stroke [47].

Changing interhemispheric interactions can promote motor-function recovery in patients with hemiplegia. Yamada et al. 2013 [48] applied fMRI and found the bilateral activation group had a significantly higher lateral index and activator voxels that transferred to the affected contralesional hemisphere after 12 low-frequency 40-min rTMS and 15-day daily occupational therapy. The unilateral activation group had a significantly increased activation in the affected contralesional hemisphere. This transfer of interhemispheric activation suggests that rTMS induces excitability changes in both hemispheres, which forms the basis of functional recovery in stroke patients.

Bashir et al. 2016 [45] found that performing 1-Hz rTMS on the affected contralesional hemisphere enhances cortical excitability on the healthy side and motor response of the hemiplegic hand more effectively than that of patients without stroke. However, another study that randomly divided 45 patients with ischemic or hemorrhagic stroke into 10-Hz rTMS, 1-Hz rTMS or sham groups showed different results. Test results in the HF-rTMS group were significantly higher than those in the LF-rTMS and sham groups. The results indicated that the unaffected contralesional hemisphere HF-rTMS’s was more effective than that of LF-rTMS and sham for motor-function recovery in patients with severe hemiplegic stroke [49]. This phenomenon is related to the two poststroke recovery mechanisms.

In other words, rTMS has a positive effect on motor-function recovery in patients with acute and chronic stroke, with a wide application value. However, the motor function of patients during different recovery periods significantly varied. With the nerve reorganization after stroke, motor function continues to recover. Therefore, the corresponding rTMS stimulation mode should be adjusted to adapt to the patients’ motor-function recovery process. Moreover, further studies should verify the effect of rTMS in different stroke patients.

### 3.4. Mechanism of rTMS in Poststroke Motor-Function Recovery

Traditionally, it has been suggested that rTMS may be useful in alleviating interhemispheric imbalance by increasing the excitability of the ipsilesional hemisphere or inhibiting the excitability of the contralesional hemisphere. The possible options of poststroke motor recovery take advantage of rTMS in regulating cortical excitability. rTMS over the motor cortex produces long-term changes in excitability. These changes promote motor recovery by regulati ng interhemispheric excitability and promoting neural plasticity [50,51]. TMS can provide relevant evidence for determining causes of stroke and identifying appropriate poststroke rehabilitation interventions, and therefore, clinicians should understand the effects of poststroke neuroplasticity.

The effect of rTMS lasted at least three months posttreatment. A single course of rTMS in the acute phase can improve upper extremity function for one year [38,52]. This follow-up effect may be related to changes in the effectiveness of synaptic connections between cortical neurons, reflecting the plasticity mechanism of the brain [53,54]. The primary motor cortex (M1)’s connection between the cerebral hemispheres affects the poststroke motor-function recovery [55]. They were using the electroencephalogram (EEG) to measure the cortical response induced by rTMS. The connection between the two hemispheres in stroke patients after rTMS was strengthened. fNIRS research verified rTMS changes in cortical excitability. Urushidani et al. 2018 [56] used fNIRS and observed a dominant activation pattern in the unaffected contralesional hemisphere 28 days after the rTMS stimulation. Bilateral activation patterns were observed 56 days after stimulation. Cortical activation spread to the unaffected contralesional hemisphere in stroke patients 109 days after initiating LF-rTMS. The LF-rTMS improved the motor function of the upper limbs in stroke patients and caused brain activation to transfer to the affected contralesional hemisphere.

The theory of competitive inhibition between each hemisphere suggests that inhibiting the unaffected contralesional hemisphere enhances the excitability of the affected side and produces motor-function recovery. Conversely, the generation of the cortical replacement pathway indicates that the brain has undergone complex recombination from the early stage of stroke, which promotes the compensatory mechanism of functional recovery. Thus, the high-frequency stimulation on the affected contralesional hemisphere enhances this compensatory mechanism. Although the motor-function recovery mechanism in stroke patients remains controversial, findings from the stroke recovery mechanism strongly support the crucial role of motor recovery in creating stroke-stimulation programs [46,57,58]. Twenty-one patients with acute stroke were recorded with MEPs on the affected and unaffected contralesional hemispheres. These patients were divided into three groups according to the MEP performance on the damaged muscle. Ten patients with TMS on both sides had poor motor recovery and were related to the size of normal MEPs. Five patients had MEPs on the healthy side that were larger than that on the affected side due to the competitive interaction. Six patients had MEPs on the affected side larger than on the healthy side, suggesting outstanding MEP recovery due to the production of cortical substitution pathways in the affected side [59].

On this basis, the bimodal balance–recovery model proposed by Di Pino et al. suggested that the integrity of the motor area and corticospinal tract in the ipsilesional hemisphere determined the manner in which the interhemispheric balance explained the recovery after stroke [60]. Therefore, it can be assumed that the recovery mechanism of stroke patients with different degrees of injury affects the changes of brain plasticity induced by rTMS.

## 4. Pitfalls and Limitations of rTMS

rTMS is a safe and effective brain-stimulation technique to determine the stroke prognosis [18]. rTMS significantly affects the excitatory and inhibitory outputs of the intracortical motor network, increasing intracortical facilitation and reducing its inhibition [61]. Although rTMS is a potential technique to restore poststroke motor function, several questions about its use in stroke remain.

Nowadays, the rTMS scheme is not standardized and a unified specific setting, i.e., location and time, of the stimulation frequency are not established. Recent studies have shown that LF-rTMS induces significant changes only in the default network, and these changes occur mainly in the stimulated hemisphere. In contrast, rTMS results in brain region changes, including salience, central executive and default networks [62]. Moreover, studies clarify that 1-Hz rTMS increases the cortical excitability in the affected hemisphere but reduces it in the unaffected hemisphere. However, 3-Hz rTMS only increases cortical excitability in the affected hemisphere [63]. The above research confirmed the important value of LF-rTMS to inhibit the contralateral cortex’s excitability in poststroke movement recovery. However, the safe application of HF-rTMS to the functional recovery among stroke patients should be further verified. Considering the selection of stimulation sites, studies on 15 patients with chronic stroke used 5-Hz rTMS to stimulate the dorsolateral prefrontal cortex (DLPFC), supplementary motor area (SMA) and M1. They found that five of them had significantly improved dual-task gait speed at posttreatment in the DLPFC target area. Therefore, whether the dominant target area is different among stroke patients should be investigated further in future studies. Moreover, the time required for the maximum–effect of rTMS should also be determined in future studies.

In addition, early evaluation and prediction of treatment effects in stroke patients is the precondition in choosing a personalized treatment plan [64,65]. As a predictor of functional recovery after stroke, MEPs that evaluate the prognosis and predict the rehabilitation of stroke patients should also consider the effects of time. Hoonhorst et al. 2018 [66] found that MEPs of the abductor digiti minimi measured within 48 h after a stroke did not affect motor function prediction. However, the best prediction appeared on the 11th day after the stroke, as characterized by MEPs of the abductor of the shoulder, abductor digiti minimi and abductor digiti minimi.

## 5. Conclusions

TMS regulation of interhemispheric inhibition imbalance is an additional therapy for poststroke motor function intervention, which has a wide range of application prospects and application values. However, the effect of interhemispheric inhibition imbalance on poststroke motor injury is still controversial. The review included only articles with conventional rTMS protocols, which limits the inference of rTMS results.

At the same time, the studies reviewed by this paper report some generic limitations. First, a small number of subjects limited the promotion of rTMS. Second, the limits of rTMS in traditional research such as the consistency of location in each experiment and the implementation effect of sham stimulation—as well as the heterogeneity of stroke subjects within the group—hinder exploration of the real effect of rTMS in the rehabilitation of stroke. Therefore, future research should combine the use of different stimulation modes based on the type of patients in order to form a standardized treatment mode through large-scale research.

## Figures and Tables

**Table 1:** Summary of transcranial magnetic stimulation (TMS) in motor-function recovery.

Author	Purpose	Number of Participants	Patient Type	Stimulation Mode	Stimulation Area	Intensity	Number of Pulses	Result
Juatmadja et al. 2020 [31]	To prove the effect of rTMS on extensor digitorum communis muscle strength improvement	18	Ischemic stroke	Affected contralesional, 10-Hz TMS	M1	100% RMT	750 pulses per day	Significant increase of sEMG numbers in the extensor digitorum communis muscle strength.
Lüdemann-Podubecká et al. 2016 [34]	To examine the effects of rTMS on hand function and cortical neurophysiology	10	Subacute stroke with mild hand motor impairment	Unaffected contralesional, 1-Hz rTMS, control-rTMS	PMd	110% RMT	900 pulses	Hand function tests revealed significant improvement of motor function of the affected, but not of the unaffected hand after actual rTMS only. Neither intervention changed the neurophysiological measures compared with those at baseline.
Long et al. 2018 [35]	To compare the effects of LF- and LF-HF rTMS on upper limb motor function	62	Upper limb hemiparesis in the early phase of stroke	Unaffected contralesional, 1-Hz rTMS, Unaffected contralesional 1-Hz rTMS + affected contralesional 10-Hz TMS, control rTMS	M1	90% RMT	1000 pulses	FMA scores and WMFT time over the baseline level were significantly increased in the LF-rTMS and LF-HF rTMS groups.
Matsuura et al. 2015 [33]	To investigate the effects of rTMS on functional recovery and electrophysiological measures	20	Acute stroke	Unaffected contralesional, 1-Hz rTMS, sham rTMS	Motor cortex	100% RMT	1200 pulses	The FMA score in the real rTMS group was significantly improved compared with that in the sham group. The PPT score of only the affected limb was improved by rTMS.
Du et al. 2016 [39]	To compare the effects of HF-rTMS versus LF-rTMS on motor recovery and identify the neurophysiological correlation of motor improvements.	69	First-ever ischemic stroke with motor deficits	Unaffected contralesional, 1-Hz rTMS, affected contralesional, 3-Hz rTMS, sham rTMS	M1	80%–90% RMT	1200 pulses	The upper limb score of FMA in the 1-Hz group was significantly improved, but no difference was observed in the other groups. The lower limb score of FMA showed significant improvements in each real rTMS group compared with that in the sham group. The MRC score in both real rTMS groups was significantly improved compared with that in the sham group.
Nam et al. 2018 [36]	To investigate the long-term effect of rTMS on improvement of motor function	76	Subacute stroke	Affected contralesional, 10-Hz TMS, control rTMS	M1	80% RMT	Repeated 20 times for a total of 1000 pulses	The motor strength, MFT, FAC classification and K-MBI scores did not differ between rTMS and control groups and rTMS did not have a long-term effect.
Hirakawa et al. 2018 [40]	To test the treatment effects of upper limb motor function	26	Chronic poststroke with severe upper limb	Unaffected contralesional, 1-Hz rTMS	Hand area of M1	90% RMT	One session consisted of 880 pulses	The FMA total score significantly increased from 12.6 to 18.0 points. The WMFT log performance time also significantly improved from 3.6 to 3.3.
Aşkın et al. 2017 [41]	To assess the efficacy of rTMS on upper extremity motor recovery and functional outcomes	40	Chronic ischemic stroke	Unaffected contralesional, 1-Hz rTMS, PT	M1	90% RMT	1200 pulses	FMA, BBT, motor and total FIM scores and FAS scores were significantly increased in both groups.
Goh et al. 2020 [42]	To investigate the effect of rTMS on improving dual-task gait performance	15	Left chronic stroke	Affected contralesional, 5-Hz rTMS	DLPFC, SMA, M1	90% RMT	1200 pulses	Single-task gait speed remained unchanged after rTMS. rTMS applied to DLPFC appeared to result in a greater change in dual-task gait speed than that at the other two sites.
Wang et al. 2019 [43]	To investigate whether HF-rTMS enhances the effects of subsequent treadmill training	14	Poststroke time longer than 6 months	Affected contralesional, 5-Hz rTMS	The motor hot spot of the tibialis anterior	90% RMT	900 pulses	FMA scores, walking speed, spatial asymmetry, TA activity at follow-up and RF activity in the experimental group after training were improved and were significantly greater than those in the control–group.
Rastgoo et al. 2016 [44]	To investigate the effect of rTMS on lower extremity (LE) spasticity, motor function and motor neuronal excitability	20	Chronic stroke	Unaffected contralesional, 1-Hz rTMS, control rTMS	Lower extremity motor area	90% RMT	1000 pulses	LE-MMAS and LE-FMA scores were improved significantly only after active rTMS and this improvement was sustained 1 week after the intervention.
Bashir et al. 2016 [45]	To investigate the effect of rTMS on motor function and motor neuronal excitability	16	Chronic stroke patients and normal subjects	Unaffected contralesional, 1-Hz rTMS, right brain 1-Hz rTMS	M1	90% RMT	1200 pulses	Muscle strength, finger tapping speed and reaction–time performance increased for the hand ipsilateral to the stimulation, but not for the hand contralateral to the stimulated side.

RMT—resting motor threshold; sEMG—surface electromyography; MFT—manual function test; FAC—functional ambulation classification; K-MBI—Korean version of the modified Barthel index; WMFT—wolf motor function test; PPT—Purdue pegboard test; BBT—box and block test; FIM—functional independence measurement; FAS—functional ambulation scale.
